# Vaccine Inoculation

**Published:** 1801-07

**Authors:** 


### VACCINE INOCULATION.

CONSCIOUS, as we are, of the benefits mankind are likely to derive from Vaccine Inoculation, and fincerely wifhing to obviate every means which can impede its progrefs; and conceiving too that no other impediment of importance can poffibly arife than from the excitement of the fpurious puftule by the choice of improper virus, we here lay before our readers fome interefting communications from Dr. Waterhoufe and others who have taken up the new practice in America. Thefe we copy from an American News-paper, but we have alfo feen a letter a letter wherein the learned Profeſſor candidly mentions, that the ſpurious caſes were excited by virus taken from a puſtule advanced too far to afford the Vaccine virus in its perfect ſtate. And this, we conceive, is preciſely the idea Dr. Jenner has laboured to diffuſe, and which, indeed, we tranſcribe from a note of his in our poſſeſſion, " That the limpid fluid ſhould be taken for the purpoſe of inoculation, as ſoon as the veſicle appears ſufficiently prominent for that purpoſe, which is commonly on the fifth or ſixth day, and that it retains its active and perfect properties until the effloreſcence is formed, which is commonly on the tenth day, and that after this its effects become uncertain, and conſequently ſhould then be no LONGER truſted to."

